# Analytic performance of ScreenFire HPV RS assay Zebra BioDome format and its potential for large-scale population HPV screening

**DOI:** 10.1186/s13027-024-00622-2

**Published:** 2024-11-29

**Authors:** Jun Wang, Godwin Imade, Alani S. Akanmu, Jonah Musa, Rose Anorlu, Yinan Zheng, Olga Garcia-Bedoya, Gloria I. Sanchez, Jerome Belinson, Kyeezu Kim, Mamoudou Maiga, Demirkan B. Gursel, Atiene S. Sagay, Folasade T. Ogunsola, Robert L. Murphy, Lifang Hou

**Affiliations:** 1 Department of Preventive Medicine, Division of Cancer Epidemiology and Prevention, Feinberg School of Medicine, Northwestern University, 680 N Lake Shore Dr, Suite 1400, Chicago, IL 60611 USA; 2 Center for Global Oncology, Robert J. Havey MD Institute for Global Health, Feinberg School of Medicine, Northwestern University, Chicago, USA; 3 Department of Obstetrics and Gynecology, College of Health Sciences, University of Jos, Jos, Nigeria; 4 Department of Hematology and Blood Transfusion, College of Medicine, Lagos University Teaching Hospital, University of Lagos, Lagos, Nigeria; 5 Department of Obstetrics and Gynecology, College of Medicine, University of Lagos, Lagos, Nigeria; 6 Division of Academic Internal Medicine and Geriatrics, Institute for Minority Health Research, University of Illinois College of Medicine, Chicago, USA; 7 Infection and Cancer Group, Faculty of Medicine, University of Antioquia, Medellin, Colombia; 8 Women’s Health Institute, Cleveland Clinic, Cleveland, USA; 9 Department of Pathology, Feinberg School of Medicine, Northwestern University, Chicago, IL USA; 10 Department of Medical Microbiology, College of Medicine, University of Lagos, Lagos, Nigeria; 11 Division of Infectious Diseases, Department of Medicine, Feinberg School of Medicine, Northwestern University, Chicago, USA; 12 Robert J. Havey MD Institute for Global Health, Northwestern University, Chicago, USA

**Keywords:** High-risk human papillomavirus (HPV), Atila SceenFire HPV risk-stratification (RS) assay, Zebra BioDome HPV assay, Isothermal amplification

## Abstract

**Background:**

Easy-to-use, rapid, scalable, high-throughput, and cost-effective HPV tests are urgently needed for low-resource settings. Atila Biosystems’ high-throughput, cost-effective, and clinically validated ScreenFire HPV Risk Stratification (RS) assay identifies 13 high risk HPV (hrHPV) in 4 groups based on their oncogenic risk (i.e., HPV16, HPV18/45, HPV31/33/35/52/58, and HPV51/59/39/56/68). The current standard format is subject to laboratory contamination, which is common for any molecular PCR test. To overcome this drawback, Atila has recently upgraded it into an innovative, contamination-free Zebra BioDome format. The contamination-free feature makes this novel assay format more suitable for large-scale community- and population-based cervical screening. This study evaluated the analytical performance of the Zebra BioDome format.

**Methods:**

We conducted a study to test the analytical performance of Zebra Biodome format in comparison to the results of using the ScreenFire HPV RS assay standard format on Biorad CFX-96 real-time PCR instrument. We used overall agreement rate and unweighted kappa value to compare the performance.

**Results:**

The overall agreement for detection of hrHPV was 96.0% with unweighted kappa value 0.94 (95% confidence interval: 0.90–0.98). The agreement rates between hrHPV genotype 16 and risk stratification genotype group (HPV18/45, HPV31/33/35/52/58, and HPV51/59/39/56/68) were all > 97.5%.

**Conclusion:**

The innovative ScreenFire HPV RS assay Zebra BioDome format produced highly concordant results with the standard format. The shared features by the two assay formats, such as easy-to-use, high throughput, cost-appropriate, and no requirements for DNA extraction. The unique contamination-prevention feature along with no requirement of preparation of reagents make the Zebra BioDome format more suitable for large-scale HPV screening to reduce global cervical cancer burden.

**Supplementary Information:**

The online version contains supplementary material available at 10.1186/s13027-024-00622-2.

## Introduction

Globally, cervical cancer is one of the most frequently diagnosed cancers and a leading cause of cancer-related deaths in women in low- and middle-income countries (LMICs) which accounts for nearly 85% of the global cervical cancer burden [1, 2]. Cervical cancer is driven by the persistence of high-risk human papillomavirus (hrHPV) infection, the most significant risk factor for the development of cervical cancer [3, 4]. The infection clears spontaneously in most cases (70-80%) but long-term, persistent infection with hrHPV increases the risk for oncogenic progression (particularly for some genotypes of the virus) [5]. This highlights the importance of population-based screening for hrHPV. In 2022, the World Health Organization (WHO) released new guidance shifting primary cervical cancer screening recommendations to HPV DNA testing in all settings, and away from unaided visual inspection using acetic acid (VIA) [6]. An affordable, easy-to-use, and high-throughput hrHPV test is urgently needed in low-resource settings, in particular LMICs, to facilitate the uptake of hrHPV DNA screening at a population level.

**Fig. 1:**
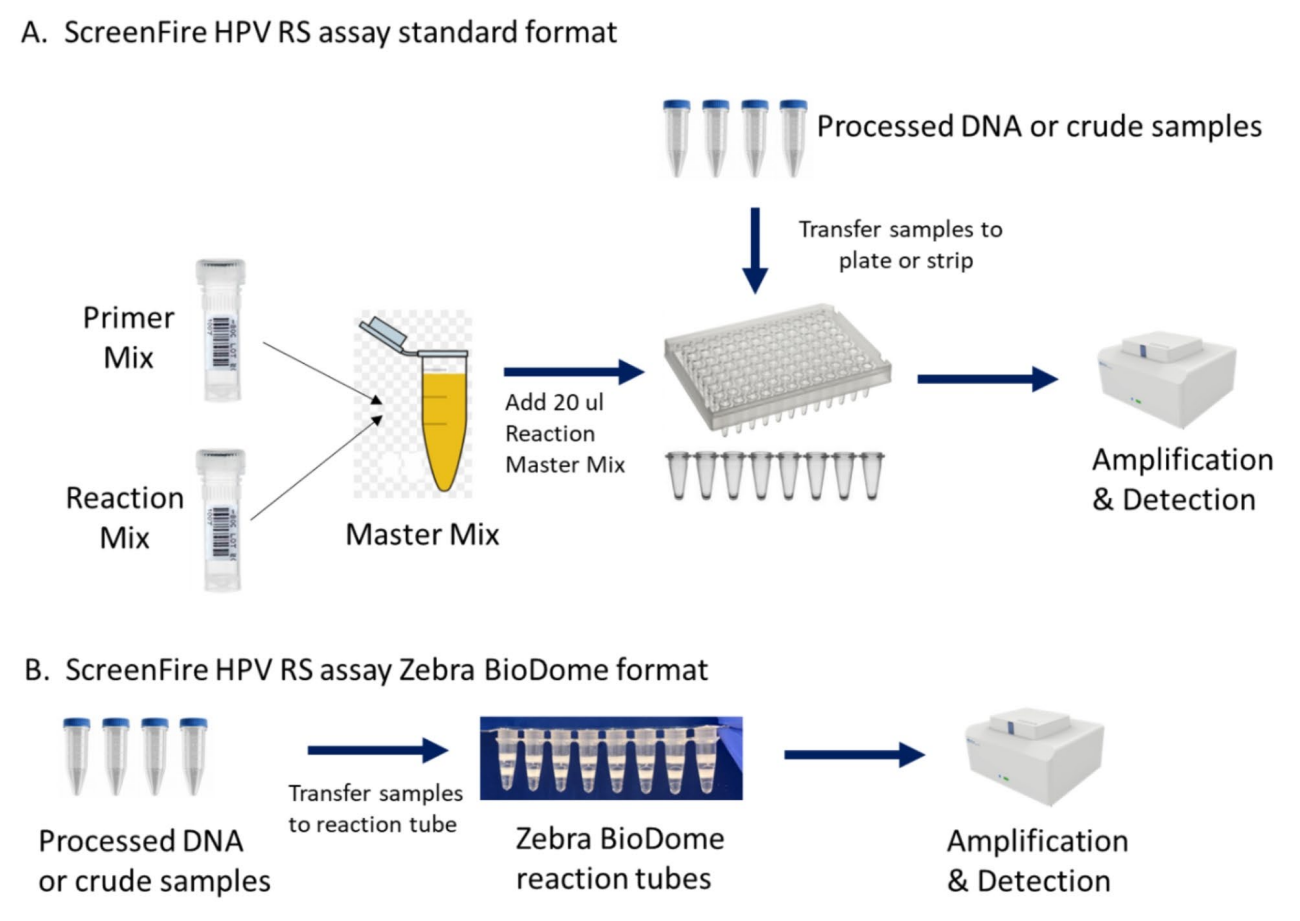
Assay flow chart of (**A**) ScreenFire HPV RS assay standard format and (**B**) ScreenFire HPV RS assay Zebre BioDome format

Current HPV genotyping assays used in LMICs face several limitations such as being time-consuming, labor-intensive, costly, or lacking high-throughput capabilities and at risk of laboratory contamination [7]. Risk of laboratory contamination, particularly common in PCR-based assays, hinders the adoption of these assays for large-scale hrHPV testing in low-resource areas without a standard laboratory setup and expert personnel. Among the existing assays, Atila Biosystems’ clinically validated ScreenFire HPV RS assay (M5FHPV-100) has been recognized as a valuable cervical cancer screening test [8–10]. It provides risk stratification (RS) genotyping information by identifying 13 high risk HPV (hrHPV) in 4 groups based on their oncogenic risk (i.e., HPV16, HPV18/45, HPV31/33/35/52/58, and HPV51/59/39/56/68) at a low cost and is high-throughput and portable, making it more feasible as a screening tool. The ScreenFire HPV RS assay uses isothermal amplification technologies and reports hrHPV in four fluorescent detection channels with channel sensitivity designed according to the order of hierarchical cancer risk factors: HPV16, HPV18/45, HPV31/33/35/52/58, and HPV39/51/56/59/68 with a sensitivity of 94.7%.[8, 9] Additionally, good concordance with other assays (e.g., AmpliTaq Gold or MY09-MY11 PCR) has been demonstrated for hrHPV detection [8]. The ScreenFire HPV RS assay also offers high capacity in standard 96-well plates with less than one hour of processing time and does not require DNA extraction and purification, a unique and important feature that makes it more suitable for primary cervical cancer screening [11]. However, the current format of the ScreenFire HPV RS assay still requires manual preparation of the master mix of reagents (Fig. 1A), needing a standard molecular laboratory setup and well-trained laboratory personnel to minimize possible laboratory contamination.

To address the challenge of laboratory contamination in HPV test with minimal lab training and setup requirements, Atila Biosystems has developed a new format for the ScreenFire HPV RS assay: the ScreenFire HPV RS assay Zebra BioDome (M5FHPV-96). This novel format features pre-loaded reagents in either 8-well PCR strips or 96-well PCR plates, covered by a temperature-sensitive hydrophobic gel matrix. The hydrogel matrix in the reagent tube is highly stable and is semi-solidified during the transportation and storage before its full implementation, which protects reagents from any possible leakage or spilling out and contamination during reagent preparation for test. The matrix liquefies and moves to the top of the liquid to seal the reaction wells during the reaction and re-solidified after amplification reactions before disposed. Thus, in addition to the common features shared by the two assay formats (e.g. easy-to-use, high-throughput, cost-appropriate in low-resource settings, and no requirements for DNA extraction), the contamination-prevention feature along with no requirement of reagent preparation make Zebra BioDome format has high potential to improve the uptake of HPV screening by requiring only the single step of adding the patient’s sample and thus making it more suitable for large-scale HPV DNA testing for cervical cancer screening. In the current study, we evaluated the analytical performance of the Zebra BioDome format in comparison with the ScreenFire standard format with the intention of using this new technology in our future large HPV DNA test implementation science studies for cervical cancer prevention in LMICs. We hereby report the first study evaluating the analytical performance of the ScreenFire HPV RS assay Zebra BioDome format compared to the clinically validated ScreenFire HPV RS assay standard format.

## Methods

### Study samples

This study is built upon our NCI-funded U54 consortium to study Epigenomic Biomarkers of HIV-Associated Cancers in Nigeria (U54CA221205). In total, 173 cervical tissue samples used in this study were collected between 2018 and 2022 in Nigerian women diagnosed with cervical cancer. The age range was 26 to 80 years old [12]. The use of these samples for this study was covered under IRB approval (STU00207051 at Northwestern University). The samples were obtained at the Jos University Teaching Hospital (JUTH) and Lagos University Teaching Hospital (LUTH) in Nigeria. DNA was extracted from the cervical biopsies using the Qiagen QIAamp DNA Mini Kit and was quantified using Qubit 4.0 fluorometer. DNA samples were stored at − 80℃ until shipment. All DNA samples were shipped in dry ice to Northwestern University and stored at − 80℃.

To further confirm that the Zebra BioDome format will perform well using crude lysed samples, we tested 46 banked vaginal swab samples with both Zebra BioDome format and the standard liquid format by the following procedures: a vaginal swab was added to a tube with lysis buffer. Then, the 5µL sample was added to two test tubes separately by following protocols for Zebra BioDome and standard format respectively. Both tubes were then put into Biorad CFX-96 real-time PCR machine for HPV test (Fig. 1A and 1B).

### Assay design features

To overcome the possible contamination hurdles faced with standard lab formats, the Zebra BioDome format is pre-packed in a PCR strip or PCR plate in a liquid format. The primer mix and enzyme mix were pre-aliquoted into the wells, but separated by the middle hydrophobic gel matrix to prevent mixing of the two reagents before samples are added for reaction (Supplemental Fig. 1A). The top gel matrix is used to keep the up-layer reagents from moving during transportation or storage and prevents reagent exposure to the environment, thereby increasing the shelf-life of the reagents. The gel matrix has lower density than water and is semi-solidified at room temperature (up to 40℃) to fix the liquid at the bottom of the wells. However, the gel matrix will liquefy and immediately move to the top of reaction mixture when the reaction starts to prevent any amplicons from leaking into the environment and contaminating the lab (Supplemental Fig. 1B).

The newly designed ScreenFire HPV RS assay Zebra BioDome format can be operated by minimally trained laboratory personnel because the operation involves only one step: adding lysed samples to pre-loaded Zebra BioDome reaction tube strips or plates with pre-aliquoted reagents. This is because the prerequisite PCR lab skills to prevent contamination and manual preparation of the master mix have been eliminated. According to the manufacturer’s unpublished data, the Zebra BioDome matrix can effectively prevent post-amplification contamination as long as the integrity of the matrix barrier is maintained. The hydrogel matrix is hydrophobic and immediately liquifies and moves to the top of the aqueous solution once the reaction starts and seals the reaction liquid from leaking into the environment. The gel matrix is resolidified after the reaction is finished, after which it is ready for disposal. Therefore, the resolidified barrier will prevent the post-amplification materials from leaking out of the tube and contaminating the environment.

### Detection and RS genotyping of hrHPV

A total of 100ng of DNA was prepared in 100 µL of 1X lysis buffer and processed to prepare the samples for hrHPV RS genotyping using both the ScreenFire HPV RS assay standard format and the ScreenFire RS assay Zebra BioDome format by Atila BioSystems, Inc (Atila, Sunnyvale, CA).

For ScreenFire HPV RS assay standard format (Fig. 1A), 20µL of freshly prepared master mix (including reaction mix and primer mix) was aliquoted into each reaction well and then a 5µL prepared DNA sample randomized and blinded in each group was added into each reaction well with master mix in it. Additionally, positive and negative controls were included in the test to monitor the test quality and laboratory contamination respectively. After the plates were sealed, vortexed gently, and centrifuged the plates were loaded into a Biorad CFX-96 real-time PCR machine. The test program was set up at 1 min per cycle at 60℃ for 60 cycles with fluorescence obtained from the following detection channels: CY5 (for HPV16), ROX (for HPV18/45), CY5.5 (for HPV31/33/35/52/58), FAM (for HPV39/51/56/59/68), and HEX (for human beta globin gene as internal control). A sample was considered positive for the corresponding HPV if the signal was detected within 60 min in the viral signal channels (FAM/ROX/Cy5/Cy5.5), regardless of the signal in the HEX channel. If no signal was detected from any of the four HPV channels within 60 min but a signal was present in the HEX fluorescent channel, then the sample was called negative. If no signal was present in any of the five fluorescent channels, the sample is invalid.

For the ScreenFire HPV RS assay Zebra BioDome format (Fig. 1B), 5 µL of the prepared DNA samples randomized and blinded in each group was directly added into the bottom of the pre-packed reaction well as specified by the manufacturer’s instructions. The sealed reaction plates were spun for 10 s to bring all liquid to the bottom then loaded into a Biorad CFX-96 real-time PCR machine. The setup of equipment was the same as described in the ScreenFire HPV RS assay standard format.

### Statistical analysis

The analytic performance of the ScreenFire HPV RS assay Zebra BioDome format was evaluated based on consistency with the ScreenFire HPV RS assay standard format according to positive, negative, and overall agreement as well as unweighted kappa values for four detection groups covering all 13 HPV genotypes (HPV16, 18, 31, 33, 35, 39, 45, 51, 52, 56, 58, 59, and 68). Detection groups were grouped hierarchically based on cervical cancer risk: HPV16 positive, else positive for HPV18/45, else positive for HPV 31/33/35/52/58, else positive for HPV51/59/39/56/68, or else negative.

## Results

Out of 173 DNA samples tested, 166 (96%) had consistent RS genotyping results between the ScreenFire HPV RS assay standard format and the ScreenFire HPV RS assay Zebra BioDome format.

On the pairwise hierarchical analysis, the two assays showed an overall agreement of 96.0% (166/173) with an unweighted kappa value of 0.94 (95% confidence interval: 0.90–0.98; Table 1). The detailed agreement rates for RS genotyping results between the two tests are shown in Table 2. The respective agreement rates between the two assay tests for HPV16, and groups of HPV18/45, and HPV31/33/35/52/58 were 97.7%, 98.8%, and 98.8% respectively and the corresponding unweighted kappa values were 0.93 (95% confidence interval 0.87-1.0), 0.96 (0.9-1.0), and 0.94 (0.86-1.0). The agreement for the group of HPV39/51/56/59/68 between the two assay tests was perfectly 100%. Thus, the ScreenFire HPV RS assay Zebra BioDome format showed highly consistent results with the ScreenFire HPV RS assay standard format.

**Table 1:** Pairwise comparison between the HPV detection results of ScreenFire HPV RS assay Zebra BioDome format (M5FHPV-96) and ScreenFire HPV RS assay standard format (M5FHPV-100) categorized hierarchically according to HPV risk groups

M5FHPV-96	M5FHPV-100					
	HPV16	HPV18/45	HPV31/33/35/52/58	HPV39/51/56/59/68	Negative	Total
**HPV16**	**36**	0	0	0	1	**37**
Row %	**97.3**	0	0	0	2.7	**100**
Column %	**92.3**	0	0	0	1.3	**21.4**
**HPV18/45**	0	**27**	0	0	1	**28**
Row %	0	**96.4**	0	0	3.6	**100**
Column %	0	**96.4**	0	0	1.3	**16.2**
**HPV31/33/35/52/58**	0	1	**18**	0	1	**20**
Row %	0	5	**90**	0	5	**100**
Column %	0	3.6	**100**	0	1.3	**11.6**
**HPV39/51/56/59/68**	0	0	0	**11**	0	**11**
Row %	0	0	0	**100**	0	**100**
Column %	0	0	0	**100**	0	**6.4**
**Negative**	3	0	0	0	**74**	**77**
Row %	3.9	0	0	0	**96.1**	**100**
Column %	7.7	0	0	0	**96.1**	**44.5**
**Total**	**39**	**28**	**18**	**11**	**77**	**173**
Row %	22.5	16.2	10.4	6.4	44.5	**100**
Column %	100	100	100	100	100	**100**

**Overall agreement rate = 96.0% (166/173); Unweighted kappa (95% CI) = 0.94 (0.90**,** 0.98)**

**Table 2:** Agreement between ScreenFire HPV RS assay Zebra BioDome format (M5FHPV-96) and ScreenFire HPV RS assay standard format (M5FHPV-100) for detection of HPV RS genotypes

HPV Results (M5FHPV-96 vs. M5FHPV-100)	+/+ *n*(%)	-/+ *n*(%)	+/- *n*(%)	-/- *n*(%)	Positive agreement % (95% CI)	Negative agreement % (95% CI)	Overall agreement % (95% CI)	Unweighted kappa (95% CI)
HPV16	36 (20.8)	3 (1.7)	1 (0.6)	133 (76.9)	92.3 (79.1–98.4)	99.3 (95.9–100)	97.7 (94.2–99.4)	0.93 (0.87-1)
HPV18/45	27 (15.6)	1 (0.6)	1 (0.6)	144 (83.2)	96.4 (81.7–99.9)	99.3 (96.2–100)	98.8 (95.9–99.9)	0.96 (0.9-1)
HPV31/33/35/52/58	18 (10.4)	0 (0)	2 (1.2)	153 (88.4)	100 (81.5–100)	98.7 (95.4–99.8)	98.8 (95.9–99.9)	0.94 (0.86-1)
HPV39/51/56/59/68	11 (6.4)	0 (0)	0 (0)	162 (93.6)	100 (71.5–100)	100 (97.7–100)	100 (97.9–100)	1 (1–1)

There were seven samples (4%) that showed different hrHPV RS genotyping results from the two assays (Supplemental Table 1A). All seven samples showed very low viral loads. We performed a limit of detection (LOD) analysis which showed these low viral loads to be near LOD and likely contributing to the inconsistent results. We therefore repeated the tests of the samples by increasing the sample input from 5µL to 10µL. The result showed 100% consistency between two tests of the seven samples (Supplemental Table 1B).

The validation results using 46 crude samples (vaginal swabs) showed that ScreenFire HPV RS assay Zebra BioDome format has generated 100% (46/46) concordant results as the ScreenFire HPV RS standard format (Table 3). The respective agreement rates between the two assay tests for the groups of HPV16, HPV18/45, HPV31/33/35/52/58 and HPV39/51/56/59/68 were all 100%.

**Table 3:** The HPV detection results of vaginal swab samples using ScreenFire HPV RS assay Zebra BioDome format (M5FHPV-96) and ScreenFire HPV RS assay standard format (M5FHPV-100) categorized hierarchically according to HPV risk groups

M5FHPV-96	M5FHPV-100					Total	Agreement
	HPV16	HPV18/45	HPV31/33/35/52/58	HPV39/51/56/59/68	Negative		
HPV16	8						100%
HPV18/45		5					100%
HPV31/33/35/52/58			13				100%
HPV39/51/56/59/68				3			100%
Negative					17		100%
Total						46	

## Discussion

In our study, when comparing the HPV RS genotype results using ScreenFire HPV RS assay standard format to those using the new ScreenFire HPV RS assay Zebra BioDome format, both percent agreement and Kappa values were high across all four hrHPV RS genotyping groups (range of 98–100% and 0.93-1.0 respectively).

Atila’s CE-in vitro diagnostic (CE-IVD) data showed that the analytical sensitivity reflected by the cycle threshold (Ct) value for hrHPV RS genotypes was below 30, although variations in Ct value exist among different HPV genotypes. The clinical significance of the large Ct value closely correlates with oncogenic risk of hrHPV genotypes. For the 4% of samples with discordant results from the two formats Ct values were greater than 30, indicating a late positive detection. The higher Ct values reflect lower HPV titers. If the viral titer in a specimen is below or close to the assay’s LOD, the test result for this specimen will become less consistent, as the probability to detect the viral target reduces. Using a 5 µL sample input has already demonstrated good concordance. However, for samples with viral loads close to the LOD, a 10 µL sample input showed improved agreement due to reduced sample variation. Therefore, Atila’s newly released protocol recommends using a 10 µL sample input in the future for the Zebra BioDome format.

A prior study comparing the lab-based ScreenFire HPV RS assay standard format to MY09-MY11 based PCR found generally excellent validity and reliability for detecting HPV [8]. The ScreenFire HPV RS assay standard format was originally developed with the goal of preventing cervical cancer in women irrespective of location globally. The hierarchical approach was designed to account for slight, potential errors in HPV detection and typing by prioritizing the typing of HPV strains that cause most cervical cancer cases (e.g., HPV16, then HPV18 and 45). This would allow countries to prioritize management resources. Questions of coinfection are avoided by organizing assay channels to reflect clinical directives based on the results of the testing, allowing for better risk stratification of women at risk of cervical cancer. In addition to the common features shared by the two assay formats (easy-to-use, high-throughput, cost-appropriate in low-resource settings, and no requirements for DNA extraction), the contamination-prevention feature along with no requirement of reagent preparation makes the Zebra BioDome format more suitable for large-scale HPV DNA testing as a primary cervical cancer screening strategy to reduce the global disparity in cervical cancer prevention and control. In addition, an affordable and portable iAMP-PS96 reader, which is now commercially available from Atila, may make the ScreenFire HPV RS assay Zebra BioDome format an even more attractive option for implementing large-scale population based hrHPV screening.

It is important to recognize that this study focused on evaluating the analytical performance of the ScreenFire HPV RS assay Zebra BioDome format. The limitations of the study include relatively small sample size and using extracted DNA. Another limitation of the current study is that we did not test enough negative samples to prove the contamination free feature of the Zebra BioDome format. To address these limitations, we did validate the Zebra BioDome format using 46 crude samples. The highly concordant results between the two formats provide solid scientific evidence demonstrating the contamination-prevention feature of the Zebro BioDome format using both extracted DNA samples and crude lysed samples. Furthermore, the contamination-free feature warrants further clinical studies in the future.

## Conclusions

The new Zebra BioDome produced highly concordant hrHPV 16 genotype and RS genotyping results compared to the ScreenFire HPV RS assay standard format. In addition to the common features shared by two assay formats (including ease-of-use, high-throughput, low cost, and no DNA extraction requirements) the contamination-prevention features make the Zebra BioDome format more suitable for large-scale population level HPV screening to enhance global cancer prevention and control efforts.

## Electronic supplementary material

Below is the link to the electronic supplementary material.

Supplementary Material 1

## Data Availability

No datasets were generated or analysed during the current study.
