# Supplementary material for: Analytic performance of ScreenFire HPV RS assay Zebra BioDome format and its potential for large-scale population HPV screening
Source: Infect Agent Cancer. 2024 Nov 29;19:59. doi: 10.1186/s13027-024-00622-2 (PMC11606105; doi:10.1186/s13027-024-00622-2)
Supplement: Supplementary file 1 — Supplementary Material 1 [file 13027_2024_622_MOESM1_ESM.docx]

**Supplemental Table 1. Samples with inconsistent results (A) and retesting results (B) from ScreenFire HPV RS assay Zebra BioDome format (M5FHPV-96) and ScreenFire HPV RS assay standard format (M5FHPV-100).**

A. Original Test

|  | **M5FHPV-100** | | **M5FHPV-96** | |
| --- | --- | --- | --- | --- |
| **Sample No.** | **HPV RS Genotype** | **Ct** | **HPV RS Genotype** | **Ct** |
| 1 | HPV16 | 33.40 | Negative | >60 |
| 2 | HPV16 | 24.86 | Negative | >60 |
| 3 | HPV16 | 48.15 | Negative | >60 |
| 4 | Negative | >60 | HPV16 | 33.10 |
| 5 | Negative | >60 | HPV18/45 | 32.01 |
| 6 | Negative | >60 | HPV31/33/35/52/58 | 33.56 |
| 7 | HPV18/45 | 41.85 | HPV31/33/35/52/58 | 37.65 |

B. Retesting of the samples

|  | **M5FHPV-100** | | **M5FHPV-96** | |
| --- | --- | --- | --- | --- |
| **Sample No.** | **HPV RS Genotype** | **Ct** | **HPV RS Genotype** | **Ct** |
| 1 | HPV16 | 51.44 | HPV16 | 30.33 |
| 2 | Negative | >60 | Negative | >60 |
| 3 | Negative | >60 | Negative | >60 |
| 4 | Negative | >60 | Negative | >60 |
| 5 | HPV18/45 | 37.66 | HPV18/45 | 44.19 |
| 6 | HPV31/33/35/52/58 | 45.06 | HPV31/33/35/52/58 | 49.14 |
| 7 | HPV18/45 | 26.57 | HPV18/45 | 28.34 |
|  | HPV31/33/35/52/58 | 25.15 | HPV31/33/35/52/58 | 28.47 |

**A**


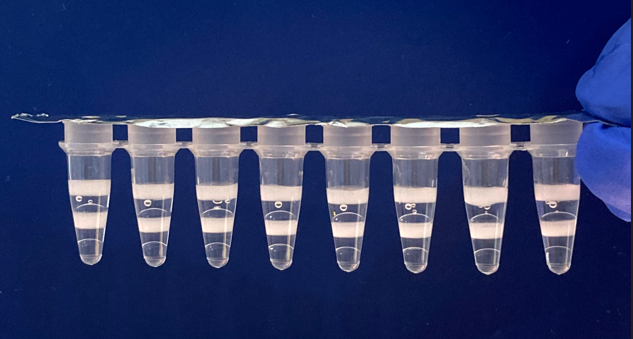


**B**


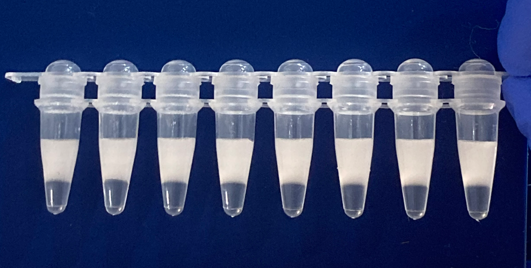


**Supplemental Figure 1. ScreenFire HPV RS assay Zebra Biodome format: A. Before the amplification: The tubes or plates are pre-packed with all test reagents. The technician just needs to add samples into the reaction wells for detection. The product's stability has been tested for a year and a half before expiration and approved as CE-IVD products (manufacturer’s data). B: After the amplification: When the reaction is initiated, the gel matrix will immediately move to the top and prevent amplicons leaking into the environment (to prevent lab contamination).**
